# The Global Burden of Disease attributable to low physical activity and its trends from 1990 to 2019: An analysis of the Global Burden of Disease study

**DOI:** 10.3389/fpubh.2022.1018866

**Published:** 2022-12-15

**Authors:** Yuan-Yi Xu, Jin Xie, Hao Yin, Fang-Fang Yang, Chun-Ming Ma, Bao-Yi Yang, Rui Wan, Bin Guo, Li-Dian Chen, Song-Lin Li

**Affiliations:** ^1^Department of Rehabilitation and Advancement, Taihe Hospital, Hubei University of Medicine, Shiyan, Hubei, China; ^2^Fujian University of Traditional Chinese Medicine, Fuzhou, Fujian, China; ^3^Taihe Hospital, Hubei University of Medicine, Shiyan, Hubei, China; ^4^Department of Intensive Care Unit, Taihe Hospital, Hubei University of Medicine, Shiyan, Hubei, China; ^5^Department of Outpatient, Taihe Hospital, Hubei University of Medicine, Shiyan, Hubei, China; ^6^Division of Financial Services, Taihe Hospital, Hubei University of Medicine, Shiyan, Hubei, China

**Keywords:** low physical activity, trends, the Global Burden of Disease, diabetes mellitus, disease attributable to low physical activity

## Abstract

**Introduction:**

Low physical activity (LPA) is associated with several major non-communicable diseases (NCDs) and premature mortality. In this study, we aimed to assess the global burden and trends in disease attributable to LPA (DALPA) from 1990 to 2019.

**Methods:**

Annual age-standardized disability-adjusted life years (DALYs) and death rates of DALPA [all-cause and five specific causes (ischaemic heart disease, diabetes mellitus, stroke, colon and rectal cancer, and breast cancer)] by sex, age, geographical region and social deprivation index (SDI) score from 1990 to 2019 were available from the Global Burden of Disease (GBD) study 2019. The estimated annual percentage changes (EAPCs) were calculated to quantify the changing trend. A generalized linear model (GLM) was used to explore the relationship between DALYs/death rates of DALPA and sociodemographic factors.

**Results:**

Globally, in 2019, the age-standardized DALYs and death rates of DALPA were 198.42/100,000 (95% UI: 108.16/100,000–360.32/100,000) and 11.10/100,000 (95% UI: 5.66/100,000–19.51/100,000), respectively. There were 15.74 million (8.51–28.61) DALYs and 0.83 million (0.43–1.47) deaths attributable to LPA. Overall, age-standardized DALYs and death rates presented significant downward trends with EAPCs [−0.68% (95% CI: −0.85– −0.50%) for DALYs and −1.00% (95% CI: −1.13– −0.86%) for deaths] from 1990 to 2019. However, age-standardized DALYs and death rates of diabetes mellitus attributable to LPA were substantially increased [EAPC: 0.76% (95% CI: 0.70–0.82%) for DALYs and 0.33% (95% CI: 0.21–0.51%) for deaths]. In the 15–49 age group, DALPA presented significant upward trends [EAPC: 0.74% (95% CI: 0.58–0.91%) for DALYs and 0.31% (95% CI: 0.1–0.51%) for deaths]. The GLM revealed that higher gross domestic product and current health expenditure (% of GDP) were negatively associated with DALYs and death rates of DALPA.

**Conclusion:**

Although global age-standardized DALYs and death rates of DALPA presented downward trends, they still cause a heavy burden worldwide. These rates showed upward trends in the diabetic and 15–49 age groups, which need more attention and health interventions.

## Introduction

Low physical activity (LPA), the most common risk factor, has been linked to several major non-communicable diseases (NCDs), including heart disease, diabetes, colon and breast cancers, mental health disorders, and premature mortality (1, 2). In 2016, the global age-standardized prevalence of insufficient physical activity was 27.5% (95% UI: 25.0–32.2) (3). In 2008, it was estimated that physical inactivity caused 9% of premature mortality or more than 5.3 million of the 57 million deaths globally (4). In the latest research, physical inactivity contributed to 7.2% of all-cause mortality, particularly in middle-income countries, in which 69% of total deaths were associated with physical inactivity (5). At the same time, physical inactivity causes a heavy economic burden worldwide. In 2013, physical inactivity caused $13.7 billion losses in global productivity (6). Healthcare costs due to physical inactivity ranged from 0.3 to 4.6% of total national healthcare expenditures (7). Currently, physical inactivity is recognized as a pandemic that requires global action (6).

However, there is still a lack of a comprehensive picture and dynamic of the global burden of disease attributable to low physical activity (DALPA), which is essential to provide valuable insights for the progress of DALPA and to guide resource allocation, program planning, and strategy development. The Global Burden of Disease (GBD) study, initiated by the World Bank and the World Health Organization, is a global comprehensive health assessment of death and disability resulting from major diseases, injuries, and related risk factors (8). The GBD 2019 provided DALPA data, including all-cause mortality and mortality from five specific causes (ischaemic heart disease, diabetes mellitus, stroke, colon and rectal cancer, and breast cancer) (9). In this study, we first quantitatively described the global burden of DALPA and then calculated the estimated annual percentage changes (EAPCs) in the age-standardized DALYs and death rates of DALPA from 1990 to 2019 at national, regional, and global scales. Finally, we used a generalized linear model (GLM) to estimate the sociodemographic factors associated with the DALYs/death rates of DALPA and sociodemographic factors. We attempted to provide a systematic understanding of DALPA.

## Methods

### Data sources

The data used in this study were included from the GBD 2019, which is maintained by ongoing multinational collaboration. The GBD 2019 provides the most up-to-date and comprehensive estimation of the global burden of 369 diseases and 87 attributable risk factors over time and by location from 1990 to 2019 (10, 11). Data including the age-standardized DALYs and death rates, as well as DALYs and deaths from DALPA with their 95% uncertainty intervals (UIs), by sex, geographical region, and SDI score from 1990 to 2019 were available from the Global Health Data Exchange GBD Results Tool (http://ghdx.healthdata.org/gbd-results-tool). Countries and territories were further classified into five SDI quintiles (low, low-middle, middle, high-middle, and high SDI scores) and 21 GBD regions (12). Age was further subdivided into three groups (15–49, 50–69, and 70+ years).

### The measure and definition of low physical activity

The Global Burden of Disease study 2019 provides a standardized and comprehensive assessment of the magnitude of risk factor exposure, relative risk, and attributable burden of disease. In the GBD 2019, two standardized questionnaires [the Global Physical Activity Questionnaire (GPAQ) and the International Physical Activity Questionnaire (IPAQ)] were used to collect self-reported physical activity information on the intensity, duration, and frequency of activities in the general adult population by random sampling (11). Then, physical activity was quantified by metabolic equivalents (METs, minutes/week), which represented the ratio of the working metabolic rate to the resting metabolic rate (13). One MET is equivalent to 1 kcal/kg/h and is equal to the energy cost of sitting quietly. According to the METs, physical activity was classified into four levels: inactive (<600 MET-minutes per week), slightly active (600–3,999 MET-minutes per week), moderately active (4,000–7,999 MET-minutes per week), and highly active (≥8,000 MET-minutes per week). LPA was defined as <3,000 MET-min per week (14). Finally, 12 separate DisMod models based on the IPAQ and GPAQ datasets were used to estimate the proportion of each country/year/age group/sex subpopulation with each of the above four activity levels, and burdens of disease attributable to LPA were estimated (11).

### The measure of disease attributable to low physical activity

The burdens of disease attributable to LPA were estimated using the comparative risk assessment framework established previously, which mainly consists of the following steps (11). First, the relative risks of low physical activity were summarized based on systematic reviews and meta-regression. Second, multiple methods were used to estimate levels of LPA for each age group/sex/location/year based on all available data sources. Third, the theoretical minimum low physical activity exposure level was defined as that associated with the lowest risk identified in published trials and cohort studies. Fourth, the attributable deaths, YLLs, YLDs, and DALYs, were computed by multiplying population attributable fractions (PAFs) by the relevant outcome quantity for each age group/sex/location/year, and the attributable burden for combinations of risk factors was also estimated.


$$\begin{array}{l} PAF_{\text{asly}}=\overset{k}{\underset{x=1}{\sum }}RR_{asy}\left(x\right)P_{\text{asly}}\left(x\right)-1/\overset{k}{\underset{x=\;1}{\sum }}RR_{as}\left(x\right)P_{\text{asly}}\left(x\right) \end{array}$$

where a is the age group, *s* is the sex, *l* is the location, and *y* is the year; PAF_asly_ is the PAF for the burden of diseases due to LPA; RR is the relative risks between exposure level x (from 1 to k) of LPA and the burden of diseases; and P is the proportion of the population exposed to low physical activity (14).

### Statistical analysis

The age-standardized DALYs and death rates were used to quantify the DALPA. The linear regression model (15) was used to assess the temporal trends of age-standardized DALYs and death rates from 1990 to 2019 at the national, regional, and global levels. The natural logarithm of age-standardized rates (ASRs) was fitted to the model, ln (ASR) = α+βX+ε, where X referred to the calendar year. The EAPC and its 95% CI were calculated as 100 × (exp(β)−1) (16). We regarded the trend as stable if the EAPC's 95% CI included 0 (*P* ≥ 0.05); otherwise, it is increased (the EAPC and its 95% CI were >0) or decreased (the EAPC and its 95% CI were <0) (17).

The generalized linear model was used to fit the DALYs and death rates of DALPA and sociodemographic factor covariates. The details are as follows: ln (DALYs or death rates) = *β*_0_ + *β*_1*_ *Gross domestic product* + *β*_2*_ *Current health expenditure (% of GDP)* + *β*_3*_ [*Population aged 60*+ *(% of total population*)] + *β*_4*_ *Urban population (% of total population)* + *β*_5*_ *Population density*. We determined the best-fitting model according to the Akaike information criterion.

## Results

### Overall burden and trends of diseases attributable to low physical activity

**Figure 1:**
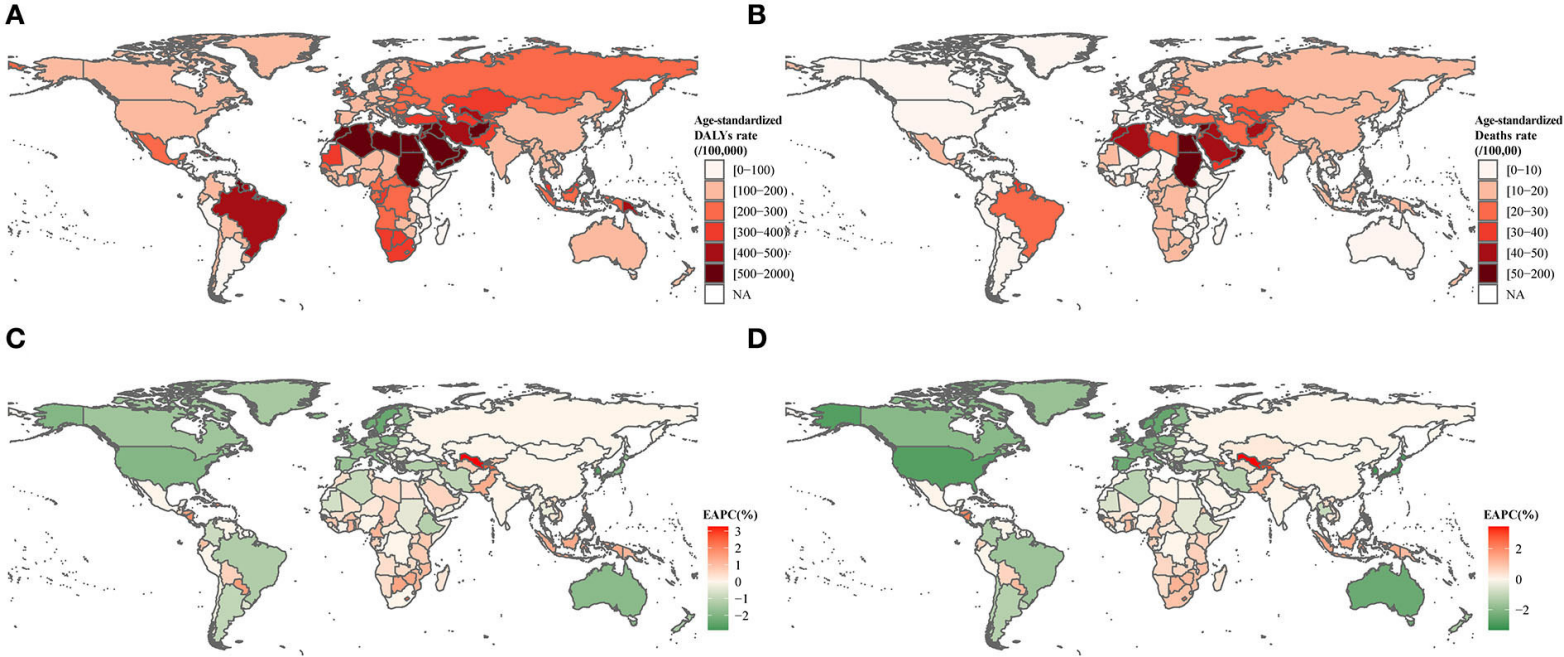
Age-standardized DALYs and death rates in 2019, EAPCs in age-standardized DALYs, and death rates from 1990 to 2019 for DALPA. **(A)** Age-standardized DALYs of DALPA in 2019. **(B)** Age-standardized death rates of DALPA in 2019. **(C)** EAPCs in age-standardized DALYs from 1990 to 2019. **(D)** EAPCs in age-standardized death rates from 1990 to 2019.

Globally, in 2019, the age-standardized DALYs and death rates of DALPA were 198.42/100,000 (95% UI: 108.16/100,000–360.32/100,000) and 11.10/100,000 (95% UI: 5.66/100,000–19.51/100,000), respectively. There were 15.74 million (8.51–28.61) DALYs and 0.83 million (0.43–1.47) deaths attributable to LPA. The burden of DALPA was higher among males than females (Table 1). The burden of DALPA was higher in countries and regions with middle and low-middle SDI scores and lower in countries with high SDI scores (Table 1). The highest age-standardized DALYs were observed in the regions of North Africa and the Middle East, Oceania, and tropical Latin America, whereas the lowest age-standardized DALYs were seen in eastern sub-Saharan Africa (Figure 1A). The age-standardized death rates demonstrated a similar geographical pattern as that of the age-standardized DALYs (Figure 1B).

**Table 1:** The number and age-standardized rate of DALYs and deaths of disease attributable to low physical activity in 1990 and in 2019.

**DALYS (95% UI)**						**Deaths (95% UI)**				
	**1990**		**2019**			**1990**		**2019**		
	**Number**	**Age-standardized rate**	**Number**	**Age-standardized rate**	**EAPC (95% CI)**	**Number**	**Age-standardized rate**	**Number**	**Age-standardized rate**	**EAPC (95% CI)**
**Global**	8,610,219 (4,279,938–15,888,866)	242.98 (122.16–463.63)	15,747,938 (8,515,094–28,617,801)	198.42 (108.16–360.32)	−0.68% (−0.85%– −0.5%)^*^	452,167 (216,170–847,600)	14.98 (7.39–27.35)	831,502 (427,076–1,470,299)	11.1 (5.66–19.51)	−1% (−1.13– −0.86%)^*^
**Gender**										
Male	3,822,568 (1,699,535–7,686,610)	242.64 (108.58–473.02)	7,393,039 (,3670,580–14,081,417)	205.53 (102.86–388.82)	−0.55% (−0.79– −0.31%)^*^	181,108 (76,631–364,253)	14.5 (6.26–28.92)	357,143 (169,396–680,789)	11.3 (5.49–21.31)	−0.82% (−0.91– −0.73%)^*^
Female	4,787,651 (2,562,970–8,529,430)	240.15 (130.11–423.46)	8,354,899 (4,779,724–14,197,377)	190.92 (109.35–324.61)	−0.77% (−0.95– −0.59%)^*^	271,060 (140,967–478,371)	15.02 (7.84–26.34)	474,359 (259,445–776,467)	10.79 (5.9–17.66)	−1.1% (−1.26– −0.94%)^*^
**Age group**										
15–49 year	845,106 (313,012–2,003,122)	31.16 (11.54–73.86)	1,511,729 (630,966–3,323,968)	38.42 (16.03–84.47)	0.74% (0.58–0.91%)^*^	14,503 (5,139–36,494)	0.53 (0.19–1.35)	22,839 (9,123–54,543)	0.58 (0.23–1.39)	0.31% (0.1–0.51%)^*^
50–69 year	3,210,061 (1,414,273–6,267,917)	470.59 (207.33–918.86)	5,498,659 (2,737,963–10,224,113)	398.76 (198.55–741.44)	−0.55% (−0.78– −0.31%)^*^	97,952 (42,020–199,610)	14.36 (6.16–29.26)	149,142 (71,519–289,033)	10.82 (5.19–20.96)	−0.95% (−1.15– −0.74%)^*^
70+ year	4,555,052 (2,241,196–8,240,767)	2,260.05 (1,112–4,088.77)	8,737,550 (4,797,272–14,984,349)	1,884.41 (1,034.62–3,231.65)	−0.61% (−0.68– −0.54%)^*^	339,713 (166,291–621,622)	168.55 (82.51–308.43)	659,522 (346,571–1,154,813)	142.24 (74.74–249.06)	−0.56% (−0.63– −0.49%)^*^
**SDI region**										
High SDI	2,484,828 (1,187,789–4,619,856)	237.33 (113.59–438.86)	2,822,705 (1,550,644–4,956,505)	148.99 (82.29–265.06)	−1.54% (−1.71%– −1.38%)^*^	153,029 (70,019–284,466)	14.81 (6.78–27.7)	160,418 (81,741–278,205)	7.11 (3.64–12.33)	−2.47% (−2.61– −2.33%)^*^
High-middle SDI	2,590,024 (1,302,702–4,906,984)	272.46 (136.82–524.66)	4,113,288 (2,253,717–7,387,863)	205.66 (113.48–369.84)	−0.93% (−1.17– −0.68%)^*^	142,712 (69,654–269,860)	17.58 (8.71–32.39)	245,873 (127,841–424,379)	12.72 (6.59–21.93)	−1.11% (−1.28– −0.95%)^*^
Middle SDI	1,971,547 (991,603–3,639,029)	223.18 (113.37–428.86)	5,116,312 (2,649,084–9,308,525)	226.37 (118.77–412.39)	0.06% (−0.06–0.19%)	89,431 (43,754–170,994)	13.03 (6.42–24.22)	253,036 (130,439–465,025)	13.08 (6.71–23.45)	0.05% (−0.14–0.23%)
Low-middle SDI	1,163,948 (597,284–2,201,058)	225.21 (118.72–408.17)	2,812,297 (150,9475–5,217,108)	227.48 (126.31–413.94)	0.03% (−0.31–0.37%)	50,340 (26,040–94,977)	12.77 (6.78–22.98)	134,393 (72,961–243,267)	12.87 (7.02–22.47)	0.05% (−0.25–0.35%)
Low SDI	392,832 (189,723–777,225)	189.88 (95.67–367)	868,376 (433,708–1,685,312)	186.9 (96.32–352.25)	−0.03% (−0.13–0.07%)	16,300 (8,006–32,465)	10.33 (5.16–19.56)	37,085 (18,670–71,177)	10.07 (5.26–18.76)	−0.04% (−0.18–0.09%)
**Type of Cause**										
Breast cancer	109,946 (51,946–202,204)	2.76 (1.3–4.99)	197,797 (97,517–345,136)	2.41 (1.18–4.18)	−0.44% (−0.65– −0.24%)^*^	4,412 (2,006–7,693)	0.12 (0.06–0.22)	8,475 (4,078–14,305)	0.11 (0.05–0.18)	−0.49% (−0.62– −0.35%)^*^
Colon and rectum cancer	497,973 (119,163–983,803)	13.58 (3.32–26.89)	1,004,854 (262,148–1,943,581)	12.57 (3.36–24.19)	−0.26% (−0.41– −0.1%)^*^	26,931 (6,792–52,678)	0.82 (0.22–1.59)	58,657 (16,866–11,2146)	0.77 (0.22–1.47)	−0.22% (−0.38– −0.05%)^*^
Diabetes mellitus	1,719,775 (782,033–3,071,194)	45 (21.34–79.47)	4,549,207 (2,188,516–7,969,495)	55.92 (27.16–97.6)	0.76% (0.7–0.82%)^*^	49,781 (24,517–84,624)	1.48 (0.75–2.46)	125,195 (62,096–20,8347)	1.62 (0.81–2.68)	0.33% (0.21–0.44%)^*^
Ischemic heart disease	4,843,158 (1,512,330–1,1108,881)	138.09 (45.07–310.37)	7,586,666 (2,613,511–16,747,205)	96.36 (33.45–210.82)	−1.21% (−1.41– −1%)^*^	283,397 (93,687–616,215)	9.46 (3.25–19.81)	486,780 (175,734–1003,279)	6.52 (2.36–13.31)	−1.34% (−1.48– −1.2%)^*^
Stroke	1,439,367 (240,415–3,930,837)	43.55 (7.56–117.17)	2,409,414 (432,866–6,377,618)	31.16 (5.69–82.02)	−1.12% (−1.29– −0.95%)^*^	87,646 (15,903–231,493)	3.09 (0.55–8.09)	152,395 (30,078–391,946)	2.08 (0.41–5.33)	−1.25% (−1.38– −1.13%)^*^
**Southeast Asia, east Asia, and Oceania**										
Southeast Asia	357,415 (159,275–728,328)	159.12 (72.36–317.48)	1,071,093 (510,119–2,015,086)	192.59 (93.69–364.18)	0.67% (0.51–0.83%)^*^	16,079 (7,272–32,907)	8.85 (4.02–17.89)	48,523 (23,525–94,810)	10.22 (4.98–19.55)	0.53% (0.37–0.69%)^*^
East Asia	989,257 (477,923–2,011,827)	146.7 (70.85–286.06)	2,608,318 (1,248,949–5,019,410)	145.71 (70.1–277.73)	−0.06% (−0.5–0.39%)	47,950 (22,371–95,799)	9.45 (4.29–18.01)	155,135 (70,839–301,472)	10.08 (4.61–19.18)	0.2% (−0.27–0.67%)
Oceania	10,606 (4,965–20,904)	384.63 (190.97–734.57)	34,526 (16,010–66,541)	512.13 (255.75–933.3)	0.96% (0.88–1.05%)^*^	380 (184–731)	18.89 (9.54–34.71)	1,211 (584–2,273)	24.26 (12.32–43.74)	0.86% (0.76–0.96%)^*^
**Sub-Saharan Africa**										
Western Sub-Saharan Africa	114,599 (51,109–231,177)	149.11 (69.02–295.01)	270,371 (128,726–524,869)	164.95 (79.63–311.33)	0.36% (0.31–0.41%)^*^	5,284 (2,411–10,757)	8.5 (3.96–16.91)	12,071 (5,835–23,512)	9.19 (4.55–17.49)	0.27% (0.23–0.3%)^*^
Central Sub-Saharan Africa	42,818 (20,060–87,679)	215.7 (103.67–423.1)	105,469 (49,307–214,533)	224.35 (110.39–447.23)	0.16% (0.05–0.26%)^*^	1,612 (761–3,246)	11.22 (5.41–21.74)	4,068 (1,956–8,143)	11.68 (5.83–22.1)	0.15% (0.06–0.23%)^*^
Southern Sub-Saharan Africa	64,099 (33,309–112,902)	238.99 (126.42–414.92)	156,452 (83,248–273,310)	300.35 (161.09–516.88)	0.78% (0.16–1.4%)^*^	2,627 (1,405–4,557)	11.69 (6.3–20.08)	6,916 (3,853–11,495)	15.82 (8.72–26.06)	1.06% (0.44–1.67%)^*^
Eastern Sub-Saharan Africa	40,573 (17,674–94,236)	61.39 (27.43–136.89)	86,497 (38,402–197,538)	61.33 (27.94–132.71)	−0.01% (−0.14–0.13%)	1,681 (754–3,806)	3.28 (1.46–7.37)	3,807 (1,721–8,300)	3.42 (1.59–6.95)	0.15% (0.05–0.25%)^*^
South Asia	1,038,360 (476,678–2,138,271)	219.98 (107.45–423.96)	2,521,754 (1,222,855–4,926,863)	205.79 (103.23–389.87)	−0.16% (−0.53–0.23%)	43,636 (20,702–86,504)	12.56 (5.97–23.69)	125,246 (62,820–234,488)	12.28 (6.2–22.24)	0.04% (−0.81–0.88%)
**Latin America and Caribbean**										
Tropical Latin America	55,9370 (321,217–893,191)	636.73 (363.81–985)	1,060,731 (674,410–1,545,535)	442.21 (282.83–640.49)	−1.23% (−1.45– −1.02%)^*^	22,780 (12,759–34,992)	32.31 (18.71–49.08)	46,039 (30,257–65,105)	20.19 (13.29–28.35)	−1.58% (−1.81– −1.35%)^*^
Caribbean	114,827 (61,351–196,442)	457.9 (245.5–773.86)	226,644 (127,559–375,365)	438.9 (247.01–726.29)	−0.1% (−0.44–0.25%)	5,731 (2,957–9,505)	25.08 (13.05–41.36)	10,732 (6,046–17,156)	20.67 (11.63–33.01)	−0.62% (−1– −0.24%)^*^
Andean Latin America	20241 (8070–44270)	106.53 (43.75–231.41)	63455 (29372–122469)	116.04 (53.66–223.2)	0.3% (0%−0.61%)^*^	1040 (394–2343)	6.2 (2.35–13.79)	3184 (1437–6338)	6.05 (2.74–11.99)	−0.01% (−0.41%−0.38%)
Central Latin America	136,849 (59,972–272,590)	169.6 (76.09–334.71)	382,135 (17,5588–746,326)	164.37 (75.53–318.25)	−0.06% (−0.43–0.31%)	5,529 (2,474–11,531)	8.14 (3.57–16.78)	16,992 (7,920–34,479)	7.67 (3.58–15.55)	−0.16% (−0.62–0.3%)
North Africa and Middle East	1,168,562 (593,062–2,079,278)	743.17 (394.66–1,269.24)	2,774,669 (1,553,821–4,605,278)	671.93 (389.1–1,089.52)	−0.34% (−0.5– −0.18%)^*^	52,083 (2,7534–88,413)	41.03 (22.62–67.17)	118,938 (68,023–192,144)	34.84 (20.38–54.4)	−0.55% (−0.79– −0.31%)^*^
**Central Europe, eastern Europe, and central Asia**										
Central Europe	389,819 (184,910–767,446)	288.02 (138.8–576.61)	449,406 (242,986–819,972)	204.02 (109.92–378.59)	−1.18% (−1.36– −1%)^*^	22,968 (1,0648–45,315)	19.11 (8.98–36.4)	29,661 (15,213–53,902)	13.16 (6.77–23.82)	−1.28% (−1.5– −1.07%)^*^
Central Asia	100,565 (44,213–204,356)	242.22 (107.49–489.48)	183,621 (83,640–372,325)	316.56 (147.06–622.36)	0.98% (0.71–1.24%)^*^	6,245 (2,746–12,505)	16.58 (7.17–32.55)	10,182 (4,528–20,044)	21.48 (9.76–41.4)	0.94% (0.54–1.35%)^*^
Eastern Europe	656,387 (286,290–1,363,223)	262.67 (116.86–546.75)	827,311 (384,730–1,655,166)	232.62 (108.15–473.82)	−0.34% (−0.74–0.07%)	42,901 (1,8957–85,790)	19.35 (8.6–37.47)	61,101 (28,476–113,768)	17.34 (8.09–32.3)	−0.23% (−0.58–0.13%)
**High-income regions**										
High-income North America	821,850 (361,036–1,628,151)	229.31 (101.56–454.13)	815,447 (401,024–1,522,883)	130.43 (64.35–247.58)	−1.9% (−2.11– −1.69%)^*^	49,867 (20,682–97,011)	13.47 (5.59–26.21)	42,882 (18,824–86,186)	6.03 (2.69–12.16)	−2.69% (−2.86– −2.51%)^*^
High-income Asia Pacific	306,541 (143,702–592,570)	164.55 (77.56–316.06)	433,585 (223,989–756,074)	90.47 (44.97–163.23)	−2.06% (−2.19– −1.93%)^*^	16,653 (7,342–31,518)	10.12 (4.43–19.16)	26,345 (12,585–46,285)	4.27 (2.09–7.5)	−2.97% (−3.14– −2.8%)^*^
Australasia	64,131 (29,997–122,576)	280.32 (130.39–533.95)	85,146 (49,256–136,839)	166.58 (95.23–272.4)	−1.75% (−1.95– −1.55%)^*^	3,948 (1,781–7,278)	18.23 (8.18–33.36)	5,318 (2,854–8,609)	9.25 (5.02–15)	−2.29% (−2.69– −1.89%)^*^
Western Europe	1,582,352 (775,460–2,881,469)	268.41 (133.23–488.25)	1,538,622 (859,919–2,565,771)	158.08 (88.48–268.59)	−1.73% (−2.06– −1.39%)^*^	101,470 (48,163–181,774)	17.25 (8.23–30.99)	100,394 (52,964–163,813)	8.61 (4.54–14.23)	−2.27% (−2.42– −2.12%)^*^
Southern Latin America	30,997 (13,366–71,216)	71.25 (30.63–164.57)	52,686 (27,393–101,807)	62.78 (32.67–121.29)	−0.37% (−0.84%−0.09%)	1,703 (689–4,055)	4.33 (1.7–10.35)	2,760 (1,379–5,396)	3.2 (1.6–6.27)	−0.93% (−1.36– −0.5%)^*^

* represents statistically significant.

During the past 30 years, the global age-standardized DALYs and death rates presented significant downward trends with the EAPCs [−0.68% (95% CI: −0.85– −0.50%) for DALYs and −1.00% (95% CI: −1.13– −0.86%) for deaths]. However, the most prominent increases in age-standardized DALYs and death rates were detected in the region of Central Asia [0.98% (95% CI: 0.71–1.24%) for DALYs and 0.94% (95% CI: 0.54–1.35%) for deaths], Oceania [0.96% (95% CI: 0.88–1.05%) for DALYs and 0.86% (95% CI: 0.76–0.96%) for deaths], and southern sub-Saharan Africa [0.78% (95% CI: 0.16–1.4%) for DALYs and 1.06% (95% CI: 0.44–1.67%) for deaths], whereas the most substantial decrease was detected in high-income regions (Figures 1C,D and Table 1).

### Impact of low physical activity on specific causes of mortality

**Figure 2:**
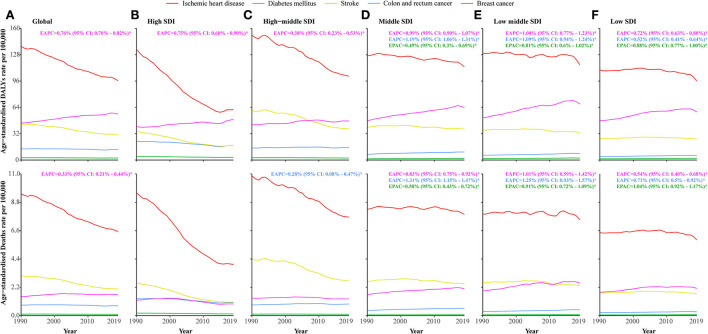
Age-standardized DALYs and death rates of specific causes attributable to low physical activity amongst SDI quintiles from 1990 to 2019. **(A)** Age-standardized DALYs and death rates of each specific cause attributable to low physical activity worldwide. **(B)** Age-standardized DALYs and death rates of each specific cause attributable to low physical activity in high SDI countries. **(C)** Age-standardized DALYs and death rates of each specific cause attributable to low physical activity in higher-middle SDI countries. **(D)** Age-standardized DALYs and death rates of each specific cause attributable to low physical activity in middle SDI countries. **(E)** Age-standardized DALYs and death rates of each specific cause attributable to low physical activity in lower-middle SDI countries. **(F)** Age-standardized DALYs and death rates of each specific cause attributable to low physical activity in low SDI countries.

Globally, age-standardized DALYs and death rates of diabetes mellitus attributable to LPA increased significantly [EAPC: 0.76% (95% CI: 0.70–0.82%) for DALYs and 0.33% (95% CI: 0.21–0.51%) for deaths], whereas those of ischaemic heart disease, stroke, colon and rectal cancer, and breast cancer decreased. Regarding SDI quintiles, the diabetes mellitus-related DALYs substantially increased at each SDI level between 1990 and 2019. Except for high and high-middle SDI levels, diabetes mellitus-related deaths also substantially increased. In regions with middle and low SDI levels, DALYs and deaths associated with breast cancer and colon and rectal cancer were substantially increased. DALYs and deaths related to ischaemic heart disease and stroke were significantly decreased at each SDI level (Figure 2 and Supplementary Table 1). The ischaemic heart disease-, diabetes mellitus-, stroke-, colon and rectal cancer-, and breast cancer-related DALYs and deaths attributable to LPA were the highest in the regions of North Africa and the Middle East, Oceania, North Africa and the Middle East, tropical Latin America, and Oceania, respectively (Supplementary Figure 1 and Supplementary Table 2).

### Disease attributable to low physical activity among the three age groups between 1990 and 2019

**Figure 3:**
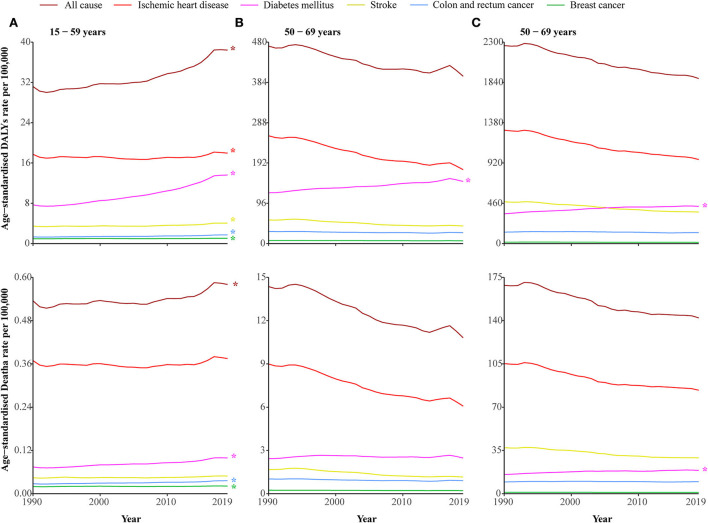
DALYs and death rates of disease attributable to low physical activity amongst the three age groups from 1990 to 2019. **(A)** 15–49 years. **(B)** 50–69 years. **(C)** 70 + years. ^*^ represents statistically significant.

Age-specific DALYs and death rates of DALPA are shown in Figure 3. Among the three age groups, elderly people had the highest DALYs and death rates (Table 1). In the 15–49 age group, DALPA showed an upward trend [0.74% (95% CI: 0.58–0.91%) for DALYs and 0.31% (95% CI: 0.1–0.91%) for deaths] (Supplementary Table 3). Notably, diabetes mellitus-related DALYs attributable to LPA were increased in all three age groups. Except among those 50–60 years old, death rates related to diabetes mellitus were increased in both the 15–49 and 70+ age groups.

### Sociodemographic factors associated with DALYs and death rates of DALPA

**Figure 4:**
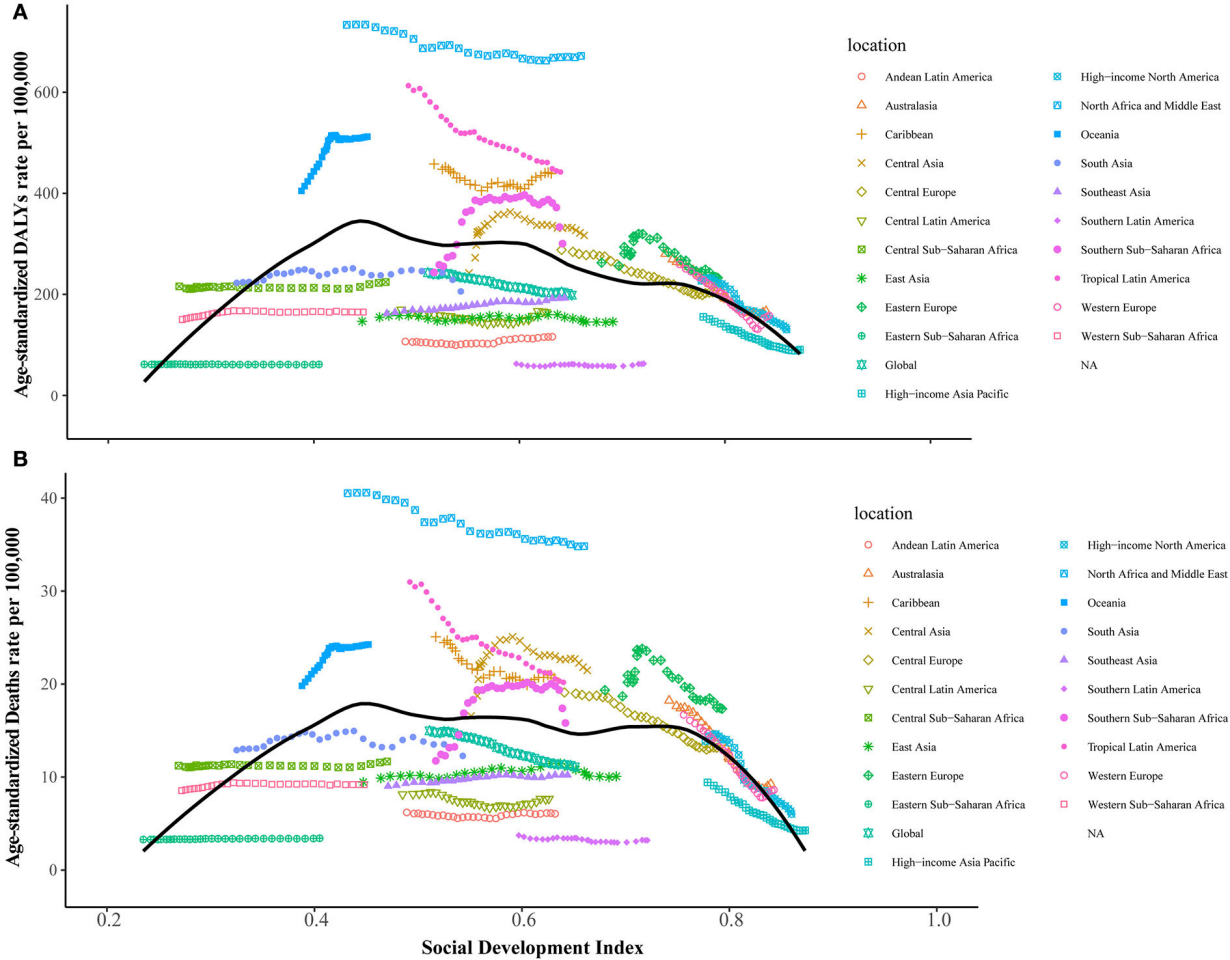
Relationships between the age-standardized DALYs and death rates of disease attributable to low physical activity and SDI level across 21 GBD regions. **(A)** Age-standardized DALYs. **(B)** Age-standardized death rates.

The generalized linear model results revealed that higher gross domestic product and current health expenditure (% of GDP) were negatively associated with DALYs and death rates of DALPA, while a greater proportion of the population aged 60+ (% of the total population) and urban population (% of the total population) were positively associated with DALYs and death rates of DALPA (Table 2). Figure 4 shows the changes in age-standardized DALYs and death rates across SDI scores by the 21 GBD regions from 1990 to 2019. Of the five regions with the highest SDI scores, all of them experienced decreases in the number of LPA-related DALYs and death rates. In contrast, from 1990 to 2019, all five regions with the lowest SDI scores experienced increases in the number of LPA-related DALYs and death rates. The associations between age-standardized DALYs and death rates and SDI scores across countries in 2019 are shown in Supplementary Figure 2.

**Table 2:** Sociodemographic factors associated with DALYs and death rates of DALPA.

	**DALYs rate**			**Death rate**		
	**Estimate**	**Std**	***P*** **-value**	**Estimate**	**Std**	***P*** **-value**
(Intercept)	5.9769	0.1703	< 0.01	2.7849	0.1627	< 0.01
Log gross domestic product	−0.0837	0.0075	< 0.01	−0.0818	0.0072	< 0.01
Current health expenditure (% of GDP)	−0.0492	0.0062	< 0.01	−0.0454	0.0059	< 0.01
Population ages 60+ (% of total population)	0.0901	0.0034	< 0.01	0.1247	0.0033	< 0.01
Urban population (% of total population)	0.0129	0.0008	< 0.01	0.0116	0.0007	< 0.01
Log population density	0.0037	0.01	0.707	−0.0122	0.0095	0.2

## Discussion

To our knowledge, this is the first comprehensive report on the disease burden attributable to LPA and its trends from 1990 to 2019 at the global, regional, and national levels. We found that global age-standardized DALYs and death rates of DALPA presented significant downward trends. However, the burden of disease was still great in the middle and low-middle SDI countries, especially in the regions of North Africa and the Middle East, Oceania, and tropical Latin America, which were the so-called hotspot regions with the highest age-standardized DALYs and death rates of DALPA in 2019. For specific causes, the age-standardized DALYs and death rates of diabetes mellitus attributable to LPA presented significant upward trends. In the 15–49 age group, the DALPA presented significant upward trends from 1990 to 2019.

In our study, we found that LPA caused a serious burden and upward trend in many middle and low-middle SDI countries. We found that the gross domestic product and current health expenditure (% of GDP) were significantly negatively associated with DALYs and death rates of DALPA. As the fourth major risk factor for many NCDs and premature mortality, a 10% reduction in the prevalence of insufficient physical activity by 2025 was recognized by the World Health Organization (WHO) as one of the global targets to improve the prevention and treatment of NCDs (18). In 2020, NCDs accounted for 80% of the global burden of disease and seven out of every 10 deaths in low- and middle-income countries (LMICs) (19). Previous studies have reported a high prevalence of inactivity among low-income and less-educated groups (20, 21). Compared to the most educated workers, workers with a primary school or no education were eight times less active (22). A possible explanation for this phenomenon is that people with a higher socioeconomic status are engaged in more leisure-time physical activity (LLPC) than people with a low socioeconomic status, who usually have insufficient resources to engage in LLPC or more actively in other domains of physical activity (23, 24). Many interventions, such as mass media campaigns, social support for physical activity within communities and worksites, and the creation and improvement of access to places for physical activity, are conducive to increasing physical activity (25). However, in LMICs, the evidence from interventions related to physical activity still has substantial gaps (26). Studies on physical activity environments and the built environment in LMICs are also insufficient, which plays a considerable role in determining the population's physical activity behaviors (27, 28). Therefore, we recommend further strengthening the relevant research to understand the correlates, determinants, and effective interventions for physical activity in LMICs, which may contribute to the global prevention of LPA.

We found that the age-standardized DALYs and death rates of diabetes mellitus attributable to LPA presented significant upward trends. The number of diabetic individuals may increase from 366 million in 2011 to 552 million by 2030 (29). A negative correlation between the risk of type 2 diabetes and physical activity has been reported in many studies (30, 31). All types of physical activity were beneficial, including leisure-time activity, vigorous activity, moderate activity, low-intensity activity, and walking (32). Regular exercise helps patients control their HbA1c and lipid levels and body composition, as well as accompanies other physical and mental benefits (33). It was suggested that patients living with a diagnosis of type 1 or type 2 diabetes should aim to engage in at least 150 min of physical activity each week, with some forms of resistance training 2 or 3 days each week (33). Diabetes mellitus is a long-term chronic disease that often necessitates medication, and patients also need to have a strong ability to manage their lifestyle and diet. Previous research showed that nearly half of patients with diabetes after diagnosis do not have good glycemic control (34); at the same time, many adults with diabetes do not meet physical activity recommendations (35). As the number of patients with diabetes increases and the poor lifestyle intensifies, the burden of diabetes mellitus attributable to LPA will continue to increase (36). In contrast, patients with the other four main causes usually have high adherence to medical advice after diagnosis, especially patients with cancer, and post-diagnosis physical activity has stronger associations with mortality risk reduction among patients with cancer (~14%) than pre-diagnosis activity (37, 38). According to our findings, the burden of diabetes mellitus-related DALYs and death rates attributable to LPA remain severe, indicating that more attention and health prevention are necessary. LPA-related DALYs and deaths significantly increased among people aged 15–49 years. The factors that affect physical activity among adults are complex. However, several factors, including community settings, systems of support, environmental circumstances, and educational level, could be modifiable to improve physical activity levels (39). Evidence has shown that increasing physical activity in later adulthood was also associated with a comparably low risk of mortality (40). Our findings show an urgent need to accelerate efforts to reduce the burden of DALPA among adults.

## Conclusion

In this study, we first revealed the global disease burden and its trends attributable to LPA. Significant downward trends were found in age-standardized DALYs and death rates of DALPA. However, the burden of disease was still great in countries and regions with middle and low-middle SDI levels. In addition, the DALYs and death rates of DALPA for diabetes mellitus and the 15–49 age group showed significant upward trends, which need more attention and health interventions.

## Data availability statement

The original contributions presented in the study are included in the article/Supplementary material, further inquiries can be directed to the corresponding author.

## Author contributions

Y-YX and S-LL designed the study and wrote the report. Y-YX, C-MM, B-YY, RW, and BG collected and analyzed the data. Y-YX, L-DC, JX, HY, F-FY, and S-LL interpreted the results. All authors revised the manuscript from the preliminary draft to submission.
