# Supplementary material for: The Global Burden of Disease attributable to low physical activity and its trends from 1990 to 2019: An analysis of the Global Burden of Disease study
Source: Front Public Health. 2022 Dec 15;10:1018866. doi: 10.3389/fpubh.2022.1018866 (PMC9798308; doi:10.3389/fpubh.2022.1018866)
Supplement: Supplementary file 1 [file Data_Sheet_1.PDF]

Supplementary Table 1. EAPCs in the age-standardised DALYs and deaths rates of specific causes attributable to low physical activity among SDI levels from 1990 to 2019.

Causes name	Index	Global	High SDI	High-middle SDI	Middle SDI	Low-middle SDI	Low SDI
**All causes**	**DALYs**	-0.68% (-0.85% - -0.5%)*	-1.54% (-1.71% - -1.38%)*	-0.93% (-1.17% - -0.68%)*	0.06% (-0.06% - 0.19%)	0.03% (-0.31% - 0.37%)	-0.03% (-0.13% - 0.07%)
	**Deaths**	-1% (-1.13% - -0.86%)*	-2.47% (-2.61% - -2.33%)*	-1.11% (-1.28% - -0.95%)*	0.05% (-0.14% - 0.23%)	0.05% (-0.25% - 0.35%)	-0.04% (-0.18% - 0.09%)
**Breast cancer**	**DALYs**	-0.44% (-0.65% - -0.24%)*	-1.12% (-1.29% - -0.96%)*	-0.46% (-0.57% - -0.35%)*	0.49% (0.3% - 0.69%)*	0.81% (0.6% - 1.02%)*	0.88% (0.77% - 1%)*
	**Deaths**	-0.49% (-0.62% - -0.35%)*	-1.23% (-1.39% - -1.08%)*	-0.24% (-0.33% - -0.14%)*	0.58% (0.43% - 0.72%)*	0.91% (0.72% - 1.09%)*	1.04% (0.92% - 1.17%)*
**Colon and rectum cancer**	**DALYs**	-0.26% (-0.41% - -0.1%)*	-0.87% (-1.1% - -0.64%)*	0.16% (-0.06% - 0.37%)	1.19% (1.06% - 1.31%)*	1.09% (0.94% - 1.24%)*	0.52% (0.41% - 0.64%)*
	**Deaths**	-0.22% (-0.38% - -0.05%)*	-0.87% (-1.01% - -0.72%)*	0.28% (0.08% - 0.47%)*	1.31% (1.15% - 1.47%)*	1.25% (0.93% - 1.57%)*	0.71% (0.5% - 0.92%)*
**Diabetes mellitus**	**DALYs**	0.76% (0.7% - 0.82%)*	0.75% (0.6% - 0.9%)*	0.38% (0.23% - 0.53%)*	0.99% (0.9% - 1.07%)*	1% (0.77% - 1.23%)*	0.72% (0.63% - 0.8%)*
	**Deaths**	0.33% (0.21% - 0.44%)*	-0.95% (-1.15% - -0.76%)*	-0.14% (-0.29% - 0.01%)	0.83% (0.75% - 0.92%)*	1.01% (0.59% - 1.42%)*	0.54% (0.4% - 0.68%)*
**Ischemic heart disease**	**DALYs**	-1.21% (-1.41% - -1%)*	-2.67% (-2.82% - -2.52%)*	-1.32% (-1.58% - -1.05%)*	-0.37% (-0.53% - -0.21%)*	-0.38% (-0.58% - -0.19%)*	-0.43% (-0.58% - -0.28%)*
	**Deaths**	-1.25% (-1.38% - -1.13%)*	-2.93% (-3.08% - -2.79%)*	-1.26% (-1.35% - -1.17%)*	-0.14% (-0.33% - 0.06%)	-0.18% (-0.56% - 0.2%)	-0.28% (-0.41% - -0.14%)*
**Stroke**	**DALYs**	-1.12% (-1.29% - -0.95%)*	-2.35% (-2.46% - -2.25%)*	-1.52% (-1.72% - -1.32%)*	-0.19% (-0.4% - 0.02%)	-0.3% (-0.52% - -0.07%)*	-0.15% (-0.27% - -0.02%)*
	**Deaths**	-1.34% (-1.48% - -1.2%)*	-2.96% (-3.1% - -2.81%)*	-1.62% (-1.78% - -1.46%)*	-0.23% (-0.42% - -0.04%)*	-0.32% (-0.56% - -0.08%)*	-0.12% (-0.26% - 0.01%)

Supplementary Table 2. EAPCs in the age-standardised DALYs and deaths rates of specific causes attributable to low physical activity among 21 GBD regions from 1990 to 2019.

	Breast cancer		Colon and rectum cancer		Diabetes mellitus		Ischemic heart disease		Stroke	
**Location name**	**DALYs**	**Deaths**	**DALYs**	**Deaths**	**DALYs**	**Deaths**	**DALYs**	**Deaths**	**DALYs**	**Deaths**
Andean Latin America	0.34% (-0.01% - 0.68%)	0.64% (0.09% - 1.19%)*	2.1% (1.63% - 2.56%)*	2.17% (1.7% - 2.65%)*	2.17% (2% - 2.33%)*	2.04% (1.76% - 2.32%)*	-0.88% (-1.11% - -0.65%)*	-0.77% (-1.09% - -0.45%)*	-0.98% (-1.34% - -0.63%)*	-0.94% (-1.41% - -0.46%)*
Australasia	-0.41% (-0.64% - -0.19%)*	-0.54% (-0.81% - -0.27%)*	-0.44% (-0.7% - -0.18%)*	-0.5% (-0.75% - -0.24%)*	1.28% (1.12% - 1.45%)*	-0.1% (-0.57% - 0.37%)	-3.24% (-3.52% - -2.96%)*	-2.99% (-3.2% - -2.78%)*	-2.46% (-2.66% - -2.27%)*	-2.5% (-3.05% - -1.95%)*
Caribbean	0.53% (0.38% - 0.68%)*	0.53% (0.37% - 0.7%)*	1.08% (0.73% - 1.43%)*	0.96% (0.58% - 1.35%)*	0.56% (0.37% - 0.76%)*	-0.15% (-0.39% - 0.09%)	-0.79% (-1.12% - -0.46%)*	-0.99% (-1.24% - -0.74%)*	-0.26% (-0.38% - -0.15%)*	-0.38% (-0.5% - -0.25%)*
Central Asia	-0.09% (-0.35% - 0.18%)	0.39% (0.25% - 0.53%)*	0.78% (0.56% - 1.01%)*	1.31% (1.04% - 1.57%)*	3.37% (3.2% - 3.54%)*	4.15% (3.73% - 4.57%)*	0.78% (0.5% - 1.05%)*	0.85% (0.49% - 1.2%)*	0.38% (0% - 0.76%)*	0.62% (0.24% - 1%)*
Central Europe	-0.05% (-0.22% - 0.12%)	0.21% (0.05% - 0.36%)*	0.63% (0.39% - 0.87%)*	0.76% (0.58% - 0.93%)*	1.15% (1.1% - 1.2%)*	0.65% (0.37% - 0.92%)*	-1.96% (-2.17% - -1.75%)*	-1.64% (-1.86% - -1.42%)*	-1.39% (-1.57% - -1.2%)*	-1.24% (-1.43% - -1.05%)*
Central Latin America	0.3% (-0.04% - 0.63%)	0.4% (0.2% - 0.61%)*	1.47% (1.15% - 1.8%)*	1.38% (1.02% - 1.74%)*	0.27% (-0.13% - 0.66%)	0.27% (-0.32% - 0.86%)	-0.41% (-0.68% - -0.14%)*	-0.31% (-0.71% - 0.1%)	-1.66% (-1.86% - -1.45%)*	-1.63% (-1.98% - -1.28%)*
Central Sub-Saharan Africa	1.1% (0.96% - 1.24%)*	1.42% (1.3% - 1.54%)*	0.33% (0.22% - 0.44%)*	0.39% (0.25% - 0.54%)*	0.42% (0.31% - 0.52%)*	0.04% (-0.08% - 0.15%)	-0.08% (-0.2% - 0.04%)	0.11% (0.01% - 0.21%)*	0.08% (0% - 0.17%)	0.28% (0.19% - 0.36%)*
East Asia	0.04% (-0.3% - 0.38%)	0.11% (-0.32% - 0.54%)	0.64% (0.28% - 1%)*	0.78% (0.56% - 1.01%)*	0.03% (-0.34% - 0.4%)	0.24% (-0.06% - 0.55%)	-0.02% (-0.54% - 0.5%)	0.43% (-0.03% - 0.89%)	-0.39% (-0.85% - 0.08%)	-0.42% (-0.95% - 0.11%)
Eastern Europe	0.2% (-0.67% - 1.08%)	0.61% (-0.3% - 1.53%)	0.47% (-0.66% - 1.61%)	0.82% (-0.17% - 1.82%)	1.41% (1.08% - 1.74%)*	2.34% (1.65% - 3.03%)*	-0.25% (-0.68% - 0.18%)	-0.25% (-0.74% - 0.25%)	-1.14% (-1.51% - -0.77%)*	-0.97% (-1.31% - -0.63%)*
Eastern Sub-Saharan Africa	0.63% (0.51% -	0.99% (0.85% -	0.75% (0.69% -	0.91% (0.86% -	-0.04% (-0.12% -	-0.02% (-0.13% -	-0.2% (-0.33% -	0% (-0.11% -	0.28% (0.22% -	0.5% (0.42% -

	0.75%)*	1.12%)*	0.82%)*	0.96%)*	0.04%)	0.09%)	-0.08%)*	0.11%)	0.35%)*	0.57%)*
High-income Asia Pacific	0.47% (0.35% - 0.6%)*	0.55% (0.34% - 0.76%)*	-0.57% (-0.73% - -0.41%)*	-0.38% (-0.68% - -0.08%)*	-0.04% (-0.15% - 0.07%)	-1.91% (-2.24% - -1.59%)*	-3.5% (-3.7% - -3.3%)*	-3.48% (-3.65% - -3.31%)*	-3.4% (-3.54% - -3.27%)*	-4.07% (-4.29% - -3.85%)*
High-income North America	-1.8% (-2.04% - -1.56%)*	-1.81% (-1.99% - -1.63%)*	-1.63% (-1.83% - -1.44%)*	-1.82% (-2.02% - -1.62%)*	0.19% (0.06% - 0.32%)*	-1% (-1.18% - -0.83%)*	-3.07% (-3.21% - -2.93%)*	-3.18% (-3.36% - -3%)*	-2.05% (-2.23% - -1.86%)*	-2.65% (-2.86% - -2.43%)*
North Africa and Middle East	1.26% (1.19% - 1.33%)*	1.29% (1.22% - 1.36%)*	1.01% (0.84% - 1.19%)*	1.03% (0.9% - 1.16%)*	1.28% (1.17% - 1.4%)*	0.3% (0.06% - 0.54%)*	-0.92% (-1.14% - -0.71%)*	-0.84% (-1.12% - -0.56%)*	0.04% (-0.09% - 0.17%)	-0.1% (-0.38% - 0.18%)
Oceania	1.1% (1.01% - 1.19%)*	1.07% (1% - 1.15%)*	0.6% (0.57% - 0.63%)*	0.66% (0.62% - 0.71%)*	1.5% (1.4% - 1.61%)*	1.38% (1.26% - 1.5%)*	0.45% (0.35% - 0.55%)*	0.52% (0.45% - 0.58%)*	0.15% (0.09% - 0.22%)*	0.16% (0.08% - 0.25%)*
South Asia	0.61% (0.17% - 1.05%)*	0.74% (0.31% - 1.17%)*	0.72% (0.28% - 1.16%)*	1.18% (0.28% - 2.09%)*	0.68% (0.2% - 1.16%)*	0.83% (-0.02% - 1.69%)	-0.5% (-0.92% - -0.08%)*	-0.09% (-1.02% - 0.86%)	-0.81% (-1.43% - -0.18%)*	-0.67% (-1.35% - 0.01%)
Southeast Asia	0.32% (0.12% - 0.53%)*	0.56% (0.34% - 0.78%)*	1.89% (1.73% - 2.04%)*	1.94% (1.8% - 2.09%)*	1.31% (1.19% - 1.43%)*	1% (0.86% - 1.15%)*	0.2% (0.06% - 0.35%)*	0.19% (0.07% - 0.31%)*	0.41% (0.28% - 0.53%)*	0.43% (0.29% - 0.56%)*
Southern Latin America	-0.03% (-0.24% - 0.18%)	0.23% (-0.04% - 0.51%)	1.36% (0.91% - 1.82%)*	1.5% (1.21% - 1.8%)*	2.09% (1.74% - 2.44%)*	1.22% (0.85% - 1.6%)*	-1.91% (-2.46% - -1.36%)*	-1.89% (-2.38% - -1.39%)*	-1.48% (-1.8% - -1.16%)*	-1.33% (-1.73% - -0.93%)*
Southern Sub-Saharan Africa	0.37% (-0.29% - 1.04%)	0.77% (0.33% - 1.21%)*	0.39% (-0.04% - 0.83%)	0.54% (0.11% - 0.97%)*	1.46% (0.96% - 1.97%)*	1.83% (1.18% - 2.49%)*	-0.04% (-0.76% - 0.69%)	0.47% (-0.18% - 1.13%)	0.47% (-0.11% - 1.06%)	0.81% (0.19% - 1.43%)*
Tropical Latin America	-0.15% (-0.31% - 0%)	-0.07% (-0.22% - 0.08%)	0.84% (0.71% - 0.98%)*	0.79% (0.65% - 0.92%)*	-0.09% (-0.22% - 0.05%)	-0.29% (-0.53% - -0.04%)*	-1.98% (-2.06% - -1.89%)*	-2.09% (-2.32% - -1.86%)*	-2.32% (-2.58% - -2.07%)*	-2.27% (-2.52% - -2.01%)*
Western Europe	-1.04% (-1.32% - -0.75%)*	-0.92% (-1.14% - -0.69%)*	-0.48% (-0.94% - -0.01%)*	-0.54% (-0.85% - -0.24%)*	0.71% (0.27% - 1.16%)*	-1.13% (-1.43% - -0.84%)*	-2.93% (-3.15% - -2.7%)*	-2.69% (-2.85% - -2.54%)*	-3.03% (-3.31% - -2.75%)*	-3.09% (-3.33% - -2.85%)*
Western Sub-Saharan Africa	1.39% (1.29% - 1.49%)*	1.42% (1.31% - 1.52%)*	1.1% (1% - 1.21%)*	1.23% (1.16% - 1.3%)*	1.11% (1.02% - 1.2%)*	1.02% (0.94% - 1.11%)*	-0.1% (-0.18% - -0.01%)*	-0.06% (-0.15% - 0.04%)	-0.16% (-0.22% - -0.1%)*	-0.09% (-0.16% - -0.02%)*

Supplementary Table 3. EAPCs in the age-standardised DALYs and deaths rates of specific causes attributable to low physical activity among the three age groups from 1990 to 2019.

Causes	Index	15-49 year	50-69 year	70+ year
**All causes**	**DALYs**	0.74% (0.58% - 0.91%)*	-0.55% (-0.78% - -0.31%)*	-0.61% (-0.68% - -0.54%)*
	**Death**	0.31% (0.1% - 0.51%)*	-0.95% (-1.15% - -0.74%)*	-0.56% (-0.63% - -0.49%)*
**Breast cancer**	**DALYs**	0.3% (0.11% - 0.49%)*	-0.42% (-0.63% - -0.22%)*	-0.41% (-0.54% - -0.28%)*
	**Death**	0.27% (0.07% - 0.47%)*	-0.5% (-0.68% - -0.32%)*	-0.24% (-0.35% - -0.12%)*
**Colon and rectum cancer**	**DALYs**	0.97% (0.62% - 1.33%)*	-0.35% (-0.62% - -0.08%)*	-0.15% (-0.31% - 0.01%)
	**Death**	0.98% (0.61% - 1.34%)*	-0.4% (-0.66% - -0.14%)*	0.06% (-0.16% - 0.29%)
**Diabetes mellitus**	**DALYs**	2.03% (1.87% - 2.18%)*	0.72% (0.55% - 0.9%)*	0.78% (0.66% - 0.9%)*
	**Death**	1.02% (0.85% - 1.19%)*	0.09% (-0.1% - 0.29%)	0.69% (0.56% - 0.82%)*
**Ischemic heart disease**	**DALYs**	0.07% (-0.14% - 0.28%)	-1.26% (-1.48% - -1.05%)*	-0.99% (-1.07% - -0.91%)*
	**Death**	0.08% (-0.13% - 0.29%)	-1.32% (-1.59% - -1.04%)*	-0.74% (-0.82% - -0.67%)*
**Stroke**	**DALYs**	0.59% (0.28% - 0.91%)*	-0.97% (-1.34% - -0.6%)*	-0.94% (-1.12% - -0.77%)*
	**Death**	0.39% (-0.04% - 0.82%)	-1.26% (-1.72% - -0.79%)*	-0.84% (-1.02% - -0.66%)*

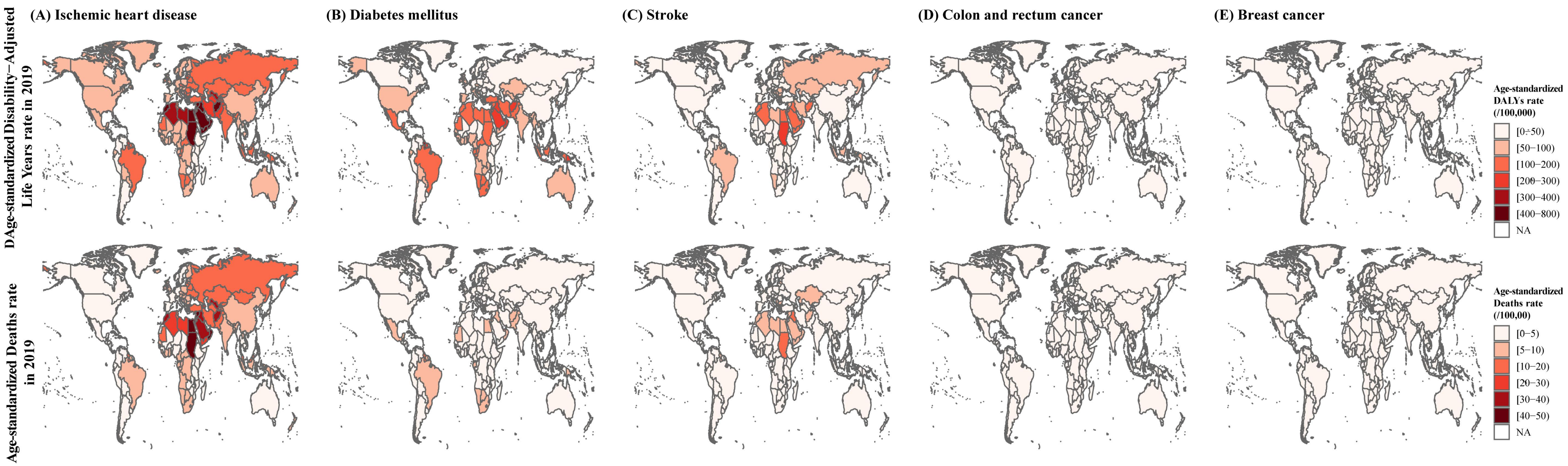

Supplementary Figure 1. **Supplementary Figure 1. Age-standardised DALYs and deaths rates of specific causes attributable to low physical activity.** (A) Ischaemic heart disease. (B) Diabetes mellitus. (C) Stroke. (D) Colon and rectal cancer. (E) Breast cancer.

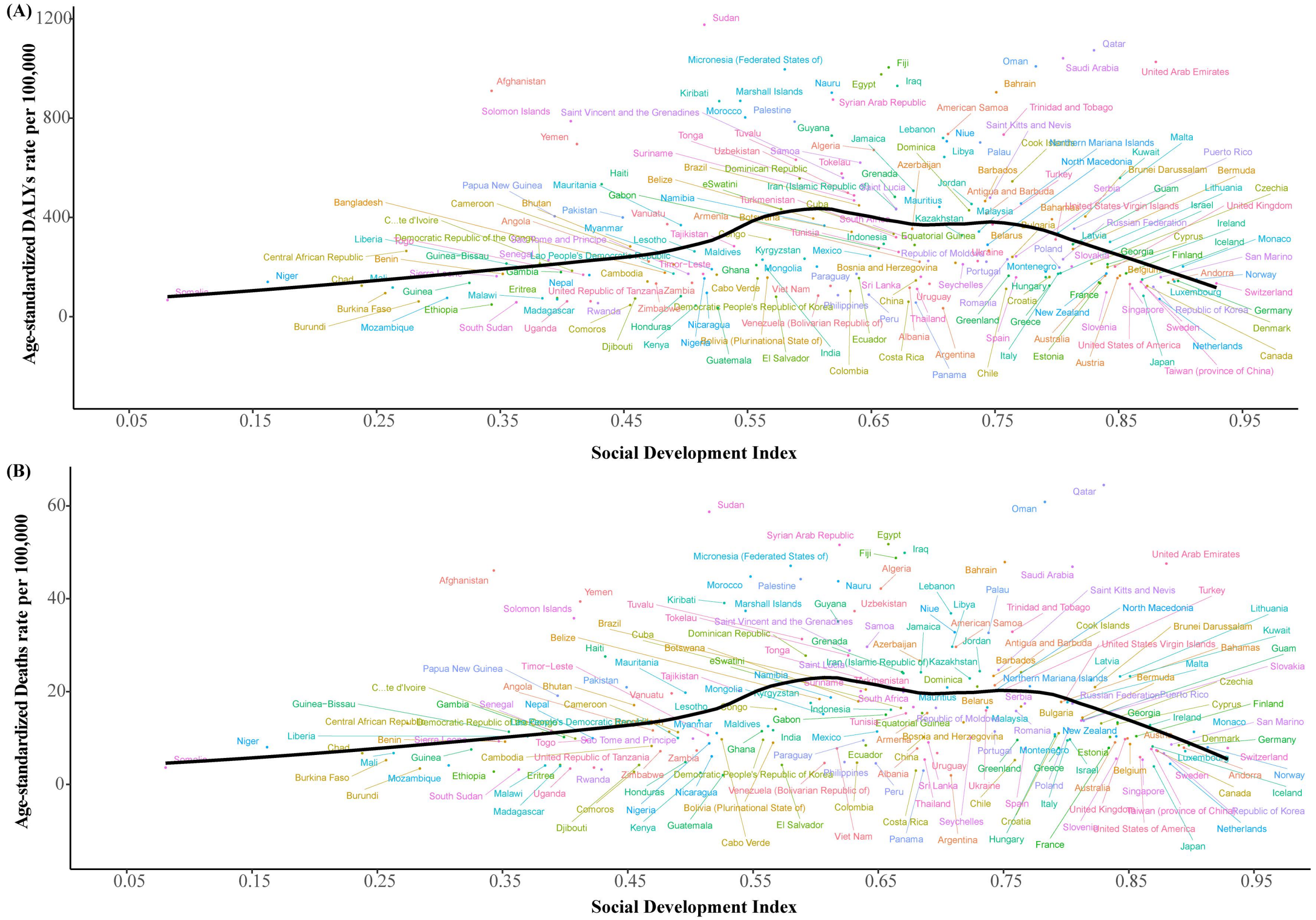

Supplementary Figure 2. **Relationships between the age-standardised DALYs and deaths rates of disease attributable to low physical activity and SDI scores across 204 countries and territories.** (A) Age-standardised DALYs. (B) Age-standardised deaths rates.
